# A matrix metalloproteinase-generated neoepitope of CRP can identify knee and multi-joint inflammation in osteoarthritis

**DOI:** 10.1186/s13075-021-02610-y

**Published:** 2021-08-31

**Authors:** Louie C. Alexander, Grant McHorse, Janet L. Huebner, Anne-Christine Bay-Jensen, Morten A. Karsdal, Virginia B. Kraus

**Affiliations:** 1 Duke Molecular Physiology Institute, Duke University School of Medicine, PO Box 104775, Carmichael Building, 300 N. Duke St, Durham, NC 27701 USA; 2 ImmunoScience, Nordic Bioscience, Herlev, Denmark; 3 Department of Medicine, Duke University School of Medicine, PO Box 104775, Carmichael Building, 300 N. Duke St, Durham, NC 27701 USA

**Keywords:** C-reactive protein (CRP), Inflammation, Synovitis, Osteoarthritis, Biomarkers

## Abstract

**Objective:**

To compare C-reactive protein (CRP) and matrix metalloproteinase-generated neoepitope of CRP (CRPM) as biomarkers of inflammation and radiographic severity in patients with knee osteoarthritis.

**Methods:**

Participants with symptomatic osteoarthritis (*n*=25) of at least one knee underwent knee radiographic imaging and radionuclide etarfolatide imaging to quantify inflammation of the knees and other appendicular joints. For purposes of statistical analysis, semi-quantitative etarfolatide and radiographic imaging scores were summed across the knees; etarfolatide scores were also summed across all joints to provide a multi-joint synovitis measure. Multiple inflammation and collagen-related biomarkers were measured by ELISA including CRP, CRPM, MMP-generated neoepitopes of type I collagen and type III collagen in serum (*n*=25), and CD163 in serum (*n*=25) and synovial fluid (*n*=18).

**Results:**

BMI was associated with CRP (*p*=0.001), but not CRPM (*p*=0.753). Adjusting for BMI, CRP was associated with radiographic knee osteophyte score (*p*=0.002), while CRPM was associated with synovitis of the knee (*p*=0.017), synovitis of multiple joints (*p*=0.008), and macrophage marker CD163 in serum (*p*=0.009) and synovial fluid (*p*=0.03). CRP correlated with MMP-generated neoepitope of type I collagen in serum (*p*=0.045), and CRPM correlated with MMP-generated neoepitope of type III collagen in serum (*p*<0.0001). No biomarkers correlated with age, knee pain, or WOMAC pain.

**Conclusions:**

To our knowledge, this is the first time that CRPM has been shown to be associated with knee and multi-joint inflammation based on objective imaging (etarfolatide) and biomarker (CD163) measures. These results demonstrate the capability of biomarker measurements to reflect complex biological processes and for neoepitope markers to more distinctly reflect acute processes than their precursor proteins. CRPM is a promising biomarker of local and systemic inflammation in knee OA that is associated with cartilage degradation and is independent of BMI. CRPM is a potential molecular biomarker alternative to etarfolatide imaging for quantitative assessment of joint inflammation.

## Background

Osteoarthritis (OA) is increasingly understood to be a disease associated with low-grade innate immune activation [1–3] warranting further studies of inflammatory markers in knee OA. C-reactive protein (CRP) is a well-studied inflammation-related molecule associated with many chronic inflammatory diseases, such as acute and chronic heart failure, coronary heart disease, and inflammatory bowel disease [4–9]. CRP has been associated with pain and decreased function in knee OA patients; however, its associations with radiographic features of OA are confounded by body mass index (BMI) [7, 10]. A prospective cohort also found that associations of CRP with incidence of knee and hip OA in women with metabolic syndrome were not significant after adjustment for BMI [11]. In the Chingford study, high serum CRP concentrations were associated with incidence of radiographic knee OA across multiple timepoints; however, this association was also not significant after adjustment for BMI [12]. In a meta-analysis of 32 studies, serum hsCRP concentrations were elevated in knee OA patients and associated with pain and decreased physical function, but were not associated with radiographic OA measures across the studies [10]. Taken together, these studies all suggest that BMI confounds associations of CRP with radiographic OA, therefore, the utility of CRP as an independent biomarker of OA, is unclear.

**Fig. 1:**
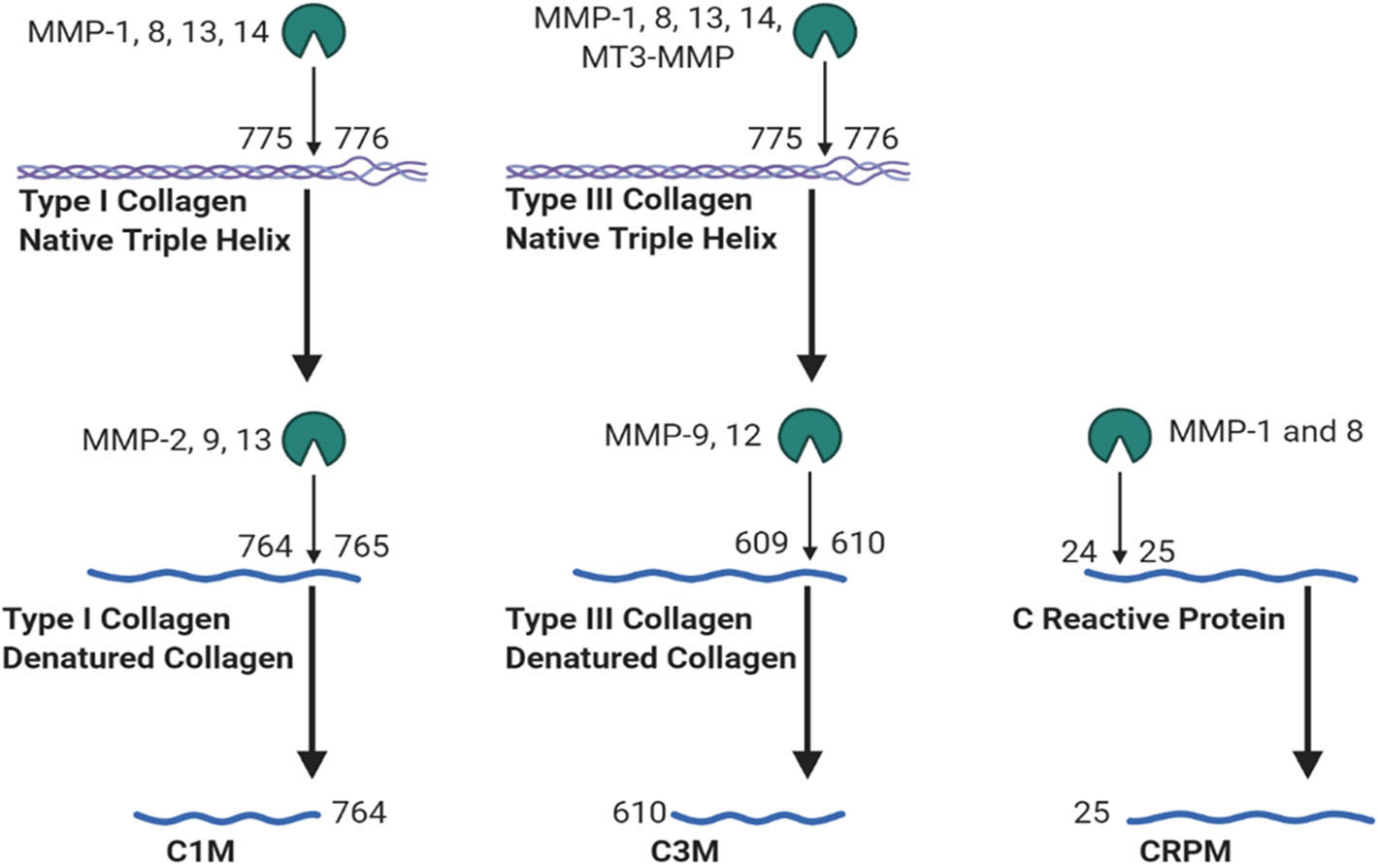
Generation of neoepitopes by MMPs*. Type I and type III collagen α1 strands are cleaved between amino acid 775 and 776 by MMPs-1, 8, 13, 14, or MT3-MMP [13]. Type I collagen fragments can be cleaved by MMP-2, 9, or 13 between amino acids 764 and 765, forming the neoepitope C1M [14]. The type III collagen fragment can be cleaved by MMP-9 or 12 between amino acids 609 and 610 to form C3M [15, 16]. CRP can be cleaved by MMP-1 or 8 between amino acid 24 and 25 to form CRPM [17]. *Figure made with https://biorender.com/

In an effort to overcome potential shortcomings of CRP, an assay has been developed to detect CRPM—a neoepitope of CRP generated by matrix metalloproteinase (MMP)-cleavage (after amino acid 25) (Fig. 1) [17]. CRP is produced in the liver, then is likely bound by Fcγ receptor II on macrophages and processed by MMPs in inflamed tissues, creating CRPM which may reflect local inflammation [17, 18]. In theory, MMP-generated neoepitope markers may be ideal OA disease activity markers because MMPs are particularly active in inflamed knees and are involved in the pathological matrix remodeling of OA. For instance, inflammatory cytokines such as IL-1, IL-6, and TNF-α activate MMPs, which cleave extracellular matrix proteins such as collagen, thereby destabilizing and degrading tissues [19]. We hypothesize that MMP-mediated cleavage products of OA-related molecules better reflect pathological turnover than their un-cleaved counterparts [20]. To test this hypothesis, we compared associations of CRPM and CRP with OA relevant indicators.

Studies to date demonstrate distinct differences between CRP and CRPM. A study comparing high-sensitivity CRP (hsCRP) and CRPM concentrations in the same cohort found that these biomarkers were not correlated; in fact, only 6% of patients (mean BMI of 31.0 kg/m^2^) had high concentrations of both markers [21]. Furthermore, in non-obese participants, 13% had high CRPM only and 12% had high hsCRP only, suggesting distinct subgroups of OA patients with elevations of each biomarker [21]. Another study of OA and rheumatoid arthritis (RA) cohorts found that serum CRPM was elevated in patients with knee OA, and moderate to high CRPM concentrations were associated with incidence of contralateral knee OA [22]. One third of these OA patients had similar CRPM concentrations to the RA cohort, suggesting a clear inflammatory phenotypic subgroup in OA [22]. High CRPM concentrations have been associated with pain sensitization in a manner independent of high sensitivity CRP and BMI [23]. In the OA LIFE study, weight loss in OA patients was associated with decreases in IL-6 and hsCRP [24]; similarly, in the IDEA exercise and diet intervention trial, weight loss was also associated with decreases in IL-6 and CRPM [25]. These findings prompted this study to investigate associations of CRPM, hsCRP, and cartilage degradation markers with localized knee and multi-joint inflammation and knee radiographic OA severity.

## Methods

### Participants

All procedures were approved by the Institutional Review Board of Duke University Medical Center. This study represents a secondary analysis of samples derived from a previously reported pilot trial (NCT01237405) to assess joint inflammation using radionuclide etarfolatide imaging in individuals (*n*=25) with radiographic knee OA [25]. Etarfolatide (99mTC-EC20) uptake through folate receptors is indicative of inflammation due to activated macrophages and neutrophils [26, 27]. In brief, 25 participants were recruited as described previously [26] based on radiographic OA (Kellgren-Lawrence (K-L) scores of 1–4) in at least one knee, 18 years or older, knee pain in the index knee on most days during 1 month of the past year. Note that participants were excluded if they had a history of immune-compromising conditions, current immune-modulating therapies, or recent surgery. The mean age was 62.4 years, mean BMI 29.2 kg/m^2^, and 72% were female. Joint pain, aching, or stiffness were assessed by National Health and Nutrition Examination Survey (NHANES) I criteria [28]; joint pain, stiffness, and daily function were assessed by the Western Ontario McMaster Osteoarthritis (WOMAC) Index [29]. Serum and plasma samples were obtained from all participants (*n*=25). Synovial fluid (SF) samples (*n*=28) available for this study were all obtained by direct aspiration from 18 individuals: 10 participants had samples from both knees, 5 from the right knee only, and 3 from the left knee only.

### Imaging

Knee radiographs were acquired using the Synaflexer frame as previously described and scored for K-L grade, joint space narrowing (JSN), and osteophyte severity (OST) using a standardized atlas by two trained readers blinded to clinical data and other imaging information with high reliability [26]. For purposes of statistical analysis, radiographic scores for both knees were summed for K-L grade (range 0–8), JSN (range 0–6), and OST (range 0–12). Etarfolatide imaging and scoring were performed as described previously [25]. For this study, intensity scores (0–3) of etarfolatide uptake were computed for 4 sites of each knee: medial and lateral synovial joint lining, and medial and lateral intra-articular sites of additional uptake indicative of localized hypertrophic synovium (score range 0–12 per knee). For purposes of statistical analysis, knee etarfolatide scores were summed for both knees (range 0–24). In addition, whole-body planar images were scored (0–3) for uptake across each of 30 joint sites: bilateral glenohumeral and acromioclavicular shoulders, elbows, wrists, hands (finger joints), thumb bases, hips, sacroiliac, medial and lateral ankles, forefoot, big toes, and sternoclavicular joints; and unilateral cervical, thoracic and lumbar spine, and manubriosternal joint. A multi-joint etarfolatide score representing multi-joint synovitis was computed by summing scores for all scored joints including both knees.

### Biomarker quantification

Sera and plasma were available for 25 participants; SF samples were available for 18 participants. Serum samples were assayed in duplicate by ELISA for the following biomarkers: C1M (Nordic Bioscience), C3M (Nordic Bioscience), high sensitivity CRP (hsCRP; MP Biomedicals, catalog#: 07BC-1119), and matrix metalloproteinase-generated CRP (CRPM; Nordic Bioscience). Mean intra-assay CVs were <3.4%, and no samples were below the limit of detection (Table 1). Assays from Nordic Bioscience were validated by the company based on the following parameters: inter- and intra-assay variation (<15%), analyte stability (<±20% variation in recovery in response to freeze-thaw, stress test, long term storage), interference (<20% in the presence of biotin, lipids, immunoglobulins, and hemolysis), and analytical specificity and sensitivity (the smallest amount of substance in a sample that can accurately be measured) for recognizing the first 6 amino acids of the neoepitopes compared to a truncated and an elongated version of that peptide with <1% displacement of the signal. The standards for each of the assays are peptides that correspond to the first 10 amino acids of the neoepitopes used to produce the monoclonal antibodies for the ELISAs [14–17]. As previously reported [30], plasma and synovial fluid samples were assayed for MMP-3, MMP-9, tissue inhibitor of matrix metalloproteinases 1 (TIMP-1), plasma CRP using the high sensitivity multiplex immunoassay Myriad Human InflammationMAP1.0 (Rules Based Medicine), and for neutrophil elastase (NE) quantified by ELISA (ThermoFisher, catalog#: BMS269, Waltham, MA) with intra-assay CVs <7%. Only two samples (8.0%) had plasma MMP-9 values below the lower limit of detection (LLOD) and thus were imputed based on an established method [31] as 1/2 LLOD; all other biomarkers had detectable concentrations. Serum CD163, plasma CD14, and SF CD163 and CD14 were quantified as reported previously [32], using Quantikine Human soluble CD163 and soluble CD14 immunoassays (R&D Systems) ELISAs; intra-assay CVs ranged from 1.2–9.2% and no values were below the LLOD.

**Table 1:** Descriptive statistics of biomarkers

*New serum biomarker measurements*			
Biomarker	Description	Median (ng/mL)	Range (ng/mL)
Serum hsCRP	Acute-phase inflammatory marker	2093.50	281.34–17,914
Serum CRPM	Degradation product of CRP	7.57	4.13–15.48
Serum C1M	Degradation product of type I collagen	33.65	14.79–58.08
Serum C3M	Degradation product of type III collagen	11.33	9.21–20.61
Serum CD163^ʌ^	Inflammatory marker of macrophages	601.70	178.40–1080.50
Plasma CD14^ʌ^	Inflammatory marker of macrophages	1.89	0.98–3.14
Plasma CRP*	Acute-phase inflammatory marker	575.00	53.80–6930.00
Plasma MMP-3*	Common MMP	7.63	3.70–68.00
Plasma MMP-9*	MMP that cleaves type I and type III collagen	12.60	ND–30.00
Plasma TIMP-1*	Inhibitor of MMP-3	36.10	24.00–54.00
Plasma MMP-3:TIMP-1	Ratio of MMP-3 to TIMP-1	0.24	0.13–1.1
SF MMP-3*	Common MMP	308	95.50–19,515.00
SF MMP-9*	MMP that cleaves type I and type III collagen	27.30	17.00–56.00
SF Neutrophil Elastase*	Neutrophil-associated protease	6842.00	1750.60–1,119,905.00
SF CD14^^^	Inflammatory marker of macrophages	809.30	178.80–1897.00
SF CD163^	Inflammatory marker of macrophages	845.00	267.50–1858.3.00

*ND* not detectable, *SF* synovial fluid

^ʌ^Previously published in Daghestani et al. 2015 (*n*=18)

*Previously published in Haraden et al. 2019 (*n*=18 for SF MMP-3 & SF MMP-9; *n*=17 for SF NE)

### Statistical analyses

For purposes of statistical analyses, knee radiographic measures and etarfolatide scores were summed for both knees per individual. For synovial fluid analyses, 1 knee per individual was selected: 8 participants had SF samples from 1 knee, 10 participants had SF samples from both knees, and 1 of these knees was randomly selected for analysis. Medians and ranges were calculated for all biomarkers, as was the ratio of plasma MMP-3 to plasma TIMP-1 (Table 1). Unadjusted Spearman correlations (GraphPad 9, San Diego, CA) were used to evaluate associations of the primary serum biomarkers (C1M, C3M, hsCRP, and CRPM) with: age; BMI; pain, aching, stiffness (PAS) NHANES I scores; WOMAC pain question 1 scores; etarfolatide uptake scores representing synovitis of both knees; total etarfolatide scores representing multi-joint synovitis; sum of knee K-L grades; sum knee OST scores; sum knee JSN scores; and concentrations of the other biomarkers (plasma MMP-3, MMP-9, TIMP-1, MMP-3/TIMP-1, CD14, and CRP; serum CD163; synovial fluid MMP-3, MMP-9, NE, CD163, and CD14). Spearman correlations of primary biomarkers with OA radiographic measures, etarfolatide knee synovitis, and multi-joint synovitis were adjusted for age, BMI, and sum K-L grade (JMP Pro 15; SAS, Cary, NC).

## Results

### hsCRP, but not CRPM, correlated with radiographic OA

Serum hsCRP, but not serum CRPM, correlated with BMI, sum K-L, and sum OST; after adjusting for age and BMI, hsCRP correlated with sum OST only (Spearman partial correlation *r*=0.505, *p*=0.014), and not sum K-L grade. Neither of these biomarkers correlated with age, PAS (pain) score, or WOMAC pain questions 1–5 (Table 2). Due to the high correlation (*r*_s_=0.99, Table 2) of serum and plasma CRP, only serum hsCRP was used for further analyses described below.

**Table 2:** Spearman correlations of biomarkers with clinical variables

	Serum hsCRP (***r***_**s**_; ***p***)	Serum CRPM (***r***_**s**_; ***p***)	Serum C1M (***r***_**s**_; ***p***)	Serum C3M (***r***_**s**_; ***p***)
Age	−0.166; 0.428	−0.108; 0.608	−0.158; 0.450	−0.089; 0.671
BMI	**0.625; 0.001**	−0.066; 0.753	0.077; 0.715	0.011; 0.959
Sum K-L	**0.453; 0.023**	−0.0639; 0.762	0.076; 0.720	−0.072; 0.734
Sum OST	**0.595; 0.002**	0.320; 0.119	0.232; 0.264	**0.417; 0.038**
Sum JSN	0.182; 0.384	−0.174; 0.407	−0.104; 0.620	−0.114; 0.586
Sum Knee Synovitis	−0.026; 0.901	**0.474; 0.017**	0.126; 0.549	0.339; 0.098
Multi-Joint Synovitis	−0.284; 0.169	**0.520; 0.008**	−0.090; 0.670	0.344; 0.092
Sum PAS knee pain	0.048; 0.819	0.049; 0.818	0.165; 0.430	0.010; 0.961
Sum WOMAC knee pain (Q1)	−0.014; 0.947	0.132; 0.530	−0.007; 0.972	0.309; 0.134

Bolded correlations were significant (*p*<0.05)

*K-L* Kellgren-Lawrence knee grade, *OST* osteophyte knee score, *JSN* joint space narrowing knee score, *PAS* pain/aching/stiffness

### CRPM, but not hsCRP, correlated with knee and multi-joint inflammation

In contrast to OA severity variables, serum CRPM, but not serum hsCRP, was correlated with knee joint inflammation as quantified by intensity of etarfolatide uptake by the knees (Table 2). Interestingly, the correlation of serum CRPM with knee synovitis was statistically significant with adjustment for age, BMI, and sum K-L grade of knees (partial Spearman correlation=0.610, *p*=0.003). In further contrast to hsCRP, serum CRPM also correlated with multi-joint synovitis (Table 2); the correlation was statistically significant adjusting for age, BMI, and sum K-L (partial Spearman correlation=0.564, *p*=0.006). Additionally, BMI correlated only with hsCRP and not CRPM or other serum biomarkers (Table 2).

### CRPM correlated strongly with MMP-cleaved type III collagen (C3M)

Underscoring the separate biological mechanisms that give rise to these biomarkers, serum hsCRP and CRPM were not correlated (Table 3). Despite the correlation of C3M and C1M, serum C3M (MMP-cleaved type III collagen) correlated significantly with serum CRPM only; in contrast, C1M (MMP-cleaved type I collagen) correlated significantly with serum hsCRP only (Table 3). Correlations of serum C3M with etarfolatide knee and multi-joint synovitis scores (*r*_s_=0.34, *p*<0.1 for both) suggest that C3M too may be predictive of synovitis (Table 2). C1M was not correlated with radiographic or etarfolatide knee scores (*p*>0.26) nor with any plasma or SF MMP concentrations (Tables 2 and 3). Interestingly, both serum CRPM and serum C3M were moderately correlated with serum and SF CD163, a marker of activated macrophages (Table 3).

**Table 3:** Spearman cross-correlations of biomarkers

	Serum hsCRP (***r***_**s**_; ***p***)	Serum CRPM (***r***_**s**_; ***p***)	Serum C1M (***r***_**s**_; ***p***)	Serum C3M (***r***_**s**_; ***p***)
Serum hsCRP	–	–	–	–
Serum CRPM	0.182; 0.385	–	–	–
Serum C1M	**0.404; 0.045**	0.358; 0.079	–	–
Serum C3M	0.202; 0.334	**0.818; <0.0001**	**0.487; 0.014**	–
Plasma CRP	**0.990; <0.0001**	0.192; 0.359	**0.423; 0.035**	0.211; 0.312
Plasma CD14	−0.021; 0.920	0.358; 0.079	0.232; 0.265	0.363; 0.074
Serum CD163	0.249; 0.231	**0.512; 0.009**	0.161; 0.443	**0.492; 0.013**
Plasma MMP-3	−0.119; 0.571	−0.130; 0.536	−0.178; 0.395	−0.181; 0.388
Plasma MMP-9	−0.152; 0.470	−0.107; 0.611	0.205; 0.325	0.096; 0.649
Plasma TIMP-1	−0.140; 0.505	−0.217; 0.297	−0.180; 0.389	−0.207; 0.321
Plasma TIMP-1: MMP-3	−0.002; 0.994	0.014; 0.948	0.007; 0.974	−0.069; 0.742
SF MMP-3*	−0.335; 0.174	0.053; 0.836	0.051; 0.842	0.129; 0.610
SF MMP-9*	0.038; 0.882	−0.118; 0.640	0.158; 0.531	−0.223; 0.375
SF Neutrophil Elastase**	−0.247; 0.337	0.061; 0.815	−0.118; 0.651	0.149; 0.567
SF CD14*	−0.233; 0.367	0.2451; 0.342	0.240; 0.352	0.248; 0.337
SF CD163*	−0.207; 0.409	**0.513; 0.030**	0.065; 0.798	**0.562; 0.015**

Bolded correlations were significant (*p*<0.05)

*SF* synovial fluid, *serum hsCRP* measured by ELISA (MP Biomedicals), *plasma CRP* measured by Luminex-based InflammationMAP 1.0 assay (RBM)

*n=18; **n=17

## Discussion

Etarfolatide imaging of the knees and multi-joint synovitis is an effective, yet complex, measure of neutrophil and macrophage activation requiring radioisotope injections [27], whereas serum CRPM—strongly correlated with etarfolatide scores—requires only a relatively simple blood draw. Furthermore, CRPM was not associated with BMI. Taken together, these results suggest that CRPM is a better disease activity and inflammation indicator in OA than CRP. Although etarfolatide imaging can in theory represent both macrophage and neutrophil infiltration of synovium, there was no correlation of CRPM with SF neutrophil elastase that is highly specific for the presence of synovial fluid neutrophils [28], suggesting that CRPM is more likely to have been generated by activated macrophages in inflamed OA joints.

Serum hsCRP, by contrast, was not associated with measures of activated macrophages, but instead was associated with BMI and C1M in this and other studies [21, 23, 24]. After adjustment for BMI, hsCRP correlated only with sum OST and not sum K-L. Few other studies have identified associations between radiographic measures and hsCRP, suggesting it lacks generalizability across different OA patient subgroups [11, 12, 24]. As an acute phase protein, CRP is generated in response to inflammation and infection and has been considered a measure of systemic inflammation; but its associations with BMI, race, heart disease, and gender confound its diagnostic capability—particularly in OA—a disease associated with gender and high BMI [7–9]. These results suggest that CRPM is an OA activity marker while hsCRP is an anatomic or structural biomarker indicating severity of radiographic OA. The unique correlations of CRPM with C3M, and of hsCRP with C1M, suggest that C3M and C1M are activity and structural markers, respectively.

**Fig. 2:**
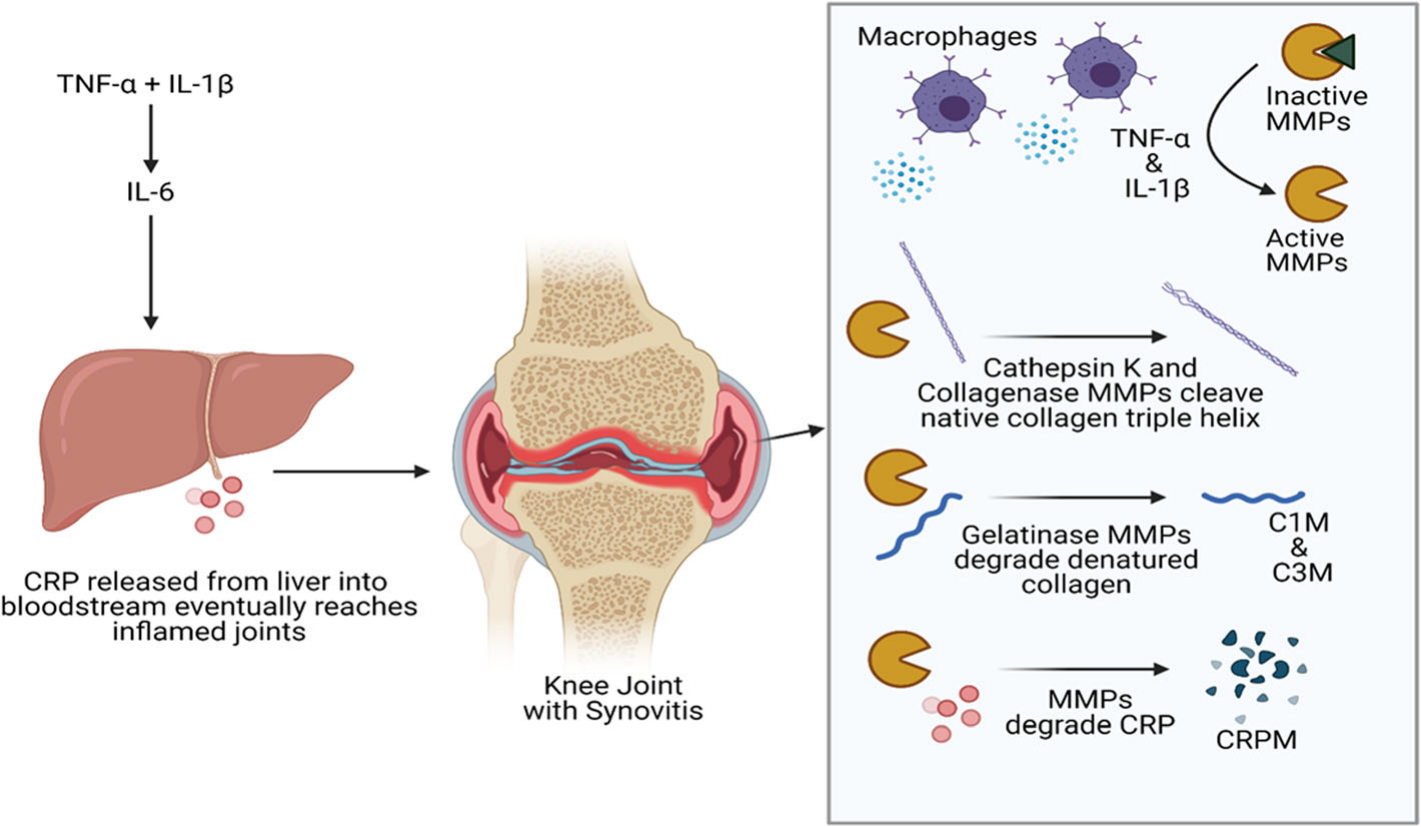
In Vivo Generation of MMP-Generated Neoepitopes in Inflamed Knee Joints*. Under inflammatory conditions, CRP is produced by the liver and released into the bloodstream [33]. In inflamed joints, MMPs are activated by inflammatory cytokines released by macrophages. These activated MMPs degrade CRP into the CRPM neoepitope [17]. Similarly, native collagen is clipped by Cathepsin K and activated Collagenase MMPs (1, 8 13, 14,) then processed by activated gelatinase MMPs [2, 9, 12, 17] to generate collagen neoepitopes (26-29;41). *Figure made with https://biorender.com/

When assessed together, the associations of these four biomarkers point to the underlying processes in inflamed synovial tissue (Fig. 2). Firstly, type I and III collagen production is upregulated by pro-inflammatory cytokines and concentrations of these collagens are elevated within osteoarthritic cartilage [34, 35]. Subsequently, these collagens are processed by MMPs into C1M and C3M, as substantiated ex vivo by pro-inflammatory cytokine treatment (tumor necrosis factor α (TNF-α) and IL-1β) of synovial membranes [34–36]. In addition, TNF-α and IL-1β induce production of IL-6, which induces production of CRP that can then be cleaved into CRPM [17, 33, 35, 36]. In fact, tocilizumab (anti-IL6 receptor antibody) treatment in RA patients significantly reduced serum CRPM in the LITHE and RADIATE trials, indicating that IL-6 is a major factor in CRPM formation [37, 38]. Furthermore, reductions in systemic CRPM—due to tocilizumab—were associated with decreased RA disease activity in RADIATE, and serum CRPM was associated with radiographic OA progression in the Rotterdam cohort [38, 39]. These findings therefore suggest that elevations of CRPM, C3M, and C1M likely reflect an active disease state of cartilage degeneration that precedes structural progression [36].

Interestingly, both serum and SF CD163—but not CD14—correlated strongly with both CRPM and C3M. Although often referred to as an M2 or anti-inflammatory marker, we showed by single-cell RNA sequencing that CD163 is also expressed by inflammatory macrophages in OA synovia in conjunction with high expression of pro-inflammatory cytokine genes including *IL1A*, *IL1B*, *IL6*, *TNF*, *CCL2,* and *CCL3* [40]. While CD14 was expressed by various macrophage types, it was also expressed on activated fibrocytes suggesting a role in wound healing. Although the reasons for the distinct associations of CRPM and C3M with CD163 are not clear at this time, these data are nevertheless consistent with the generation of CRPM and C3M by a macrophage-related process in OA joints.

This study was limited by a small sample size (*n*=25 participants) with a small number of directly aspirated synovial fluid samples (*n*=18). Unlike hsCRP, CRPM does not appear to be confounded by BMI; however, this cohort had a mean BMI of 29.2 kg/m^2^ (range 22.49–38.40) and the association of CRPM with knee OA inflammation should be evaluated in a larger cohort with a higher mean and median BMI. We previously identified associations of knee synovitis, measured by etarfolatide imaging, with synovial fluid MMP-3 and TIMP-1, suggesting these markers represent localized inflammation [40]; however, these markers were not associated with CRPM. This might be explained by the fact that the MMP analyses in this study were measures of total protein and not MMP activity that would be expected to more robustly correlate with MMP-generated epitopes. In addition, the study may have lacked sufficient power to detect a correlation due to small sample size. As our goal was to qualify biomarkers of OA inflammation using easily accessible biofluids, we focused on systemic (serum) CRPM, CRP, C1M, and C3M assays; we did not analyze any of these markers in synovial fluid.

## Conclusions

In summary, to our knowledge, this is the first time that CRPM has been shown to be associated with knee and multi-joint inflammation based on objective imaging (etarfolatide) and biomarker (CD163) measures. Furthermore, serum CRPM associations with knee and multi-joint OA-related synovitis are independent of BMI. The correlation of serum CRPM with serum and SF CD163 supports the generation of CRPM by MMPs under conditions of chronic macrophage-related inflammation in OA. In contrast, serum CRP was associated with both BMI and radiographic knee osteophyte severity. These results demonstrate the capability of biomarker measurements to reflect complex biological processes and for neoepitope markers to more distinctly reflect acute processes than their precursor proteins. In conclusion, CRPM represents a potential molecular biomarker alternative to etarfolatide imaging for quantitative assessment of joint inflammation in OA in the absence of other inflammatory conditions. In addition, given that CRPM is generated by MMP-1 and MMP-8 during inflammation, CRPM may be useful as an activity marker of inflammation in other tissues.

## Data Availability

The datasets used and analyzed during the current study are available from the corresponding author on reasonable request.

## Abbreviations

CRP: C-reactive protein
hsCRP: High-sensitivity CRP
MMP: Matrix metalloproteinase
CRPM: MMP-generated neoepitope of CRP
C1M: MMP-generated neoepitope of type I collagen
C3M: MMP-generated neoepitope of type III collagen
OA: Osteoarthritis
IL: Interleukin
K-L: Kellgren-Lawrence
NHANES: National Health and Nutrition Examination Survey
PAS: pain, aching, stiffness
WOMAC: Western Ontario McMaster Osteoarthritis
JSN: Joint space narrowing
OST: Osteophyte severity
LLOD: Lower limit of detection
TIMP-1: Tissue inhibitor of matrix metalloproteinases 1
NE: Neutrophil elastase
SF: Synovial fluid
TNF-α: Tumor necrosis factor α
